# COVID-19 mitigation for high-risk populations in Springfield Massachusetts USA: a health systems approach

**DOI:** 10.1186/s12939-021-01567-3

**Published:** 2021-10-19

**Authors:** Paul A. Pirraglia, Cristina Huebner Torres, Jessica Collins, Jane Garb, Marian Kent, Sarah Perez McAdoo, Yemisi Oloruntola-Coates, Jacob M. Smith, Abraham Thomas

**Affiliations:** 1 Baystate Health, Springfield, USA; 2 BeHealthy Partnership, Springfield, USA; 3 University of Massachusetts Chan Medical School-Baystate, Springfield, USA; 4 Caring Health Center, Springfield, USA; 5 University of Massachusetts, Amherst, USA; 6 Public Health Institute of Western Massachusetts, Springfield, USA

**Keywords:** COVID 19, Populations, vulnerable, Risk factors, Inequalities, health care, Needs Assessment, Healthcare, Community Outreach

## Abstract

**Background:**

Numerous reports have demonstrated the disproportionate impact that COVID-19 has had on vulnerable populations. Our purpose is to describe our health care system’s response to this impact.

**Methods:**

We convened a Workgroup with the goal to mitigate the impact of COVID-19 on the most medically vulnerable people in Springfield, Massachusetts, USA, particularly those with significant social needs. We did this through (1) identifying vulnerable patients in high-need geographic areas, (2) developing and implementing a needs assessment/outreach tool tailored to meet cultural, linguistic and religious backgrounds, (3) surveying pharmacies for access to medication delivery, (4) gathering information about sources of food delivery, groceries and/or prepared food, (5) gathering information about means of travel, and (6) assessing need for testing. We then combined these six elements into a patient-oriented branch and a community outreach/engagement branch.

**Conclusions:**

Our highly intentional and methodical approach to patient and community outreach with a strong geographic component has led to fruitful efforts in COVID-19 mitigation. Our patient-level outreach engages our health centers’ clinical teams, particularly community health workers, and is providing the direct benefit of material and service resources for our at-risk patients and their families. Our community efforts leveraged existing relationships and created new partnerships that continue to inform us—healthcare entities, healthcare employees, and clinical teams—so that we can grow and learn in order to authentically build trust and engagement.

## Background

The COVID-19 pandemic has emerged as a crisis within a crisis. People who were already suffering due to inequities related to their vulnerabilities are now also at highest risk from COVID-19 in terms of cases, hospitalizations, and death. Vulnerability is a broad concept with socioeconomic, racial, ethnic, and identification factors as well as medical comorbidity all contributing [1–5]. Numerous reports have demonstrated the disproportionate impact that COVID-19 has had on vulnerable populations [6–11].

Springfield, Massachusetts (MA), USA, is home to a large number of people who would be considered vulnerable. This is by virtue of being a diverse, multi-ethnic city in which people of color represent the majority of the population at 67%. Approximately 44% of residents identify as Latinx, 19% as Black, and 2% Asian. Springfield also is home for a large immigrant population, with more than 10% of residents born outside the US and 16% having migrated to Springfield from Puerto Rico [12]. Springfield has a 30% poverty rate with a total of 21,600 households whose income is under $25,000/year [13].

Effectively mitigating the inequitable impact of COVID-19 on vulnerable populations is critical, and specific tactics have been suggested [10, 11]. We have acted and continue to act upon many of the tactics that have been proposed and can offer our experience with implementing our COVID-19 mitigation program in Springfield, MA, as an example of a health system’s response to addressing inequities driven by COVID-19. Therefore, our purpose is to describe our systematic response with the intent that implementing some or all aspects of our approach may be highly applicable and adoptable elsewhere. We will describe the inception of our COVID-19 Mitigation Workgroup, the Workgroup’s objectives and goals, the structure of the Workgroup’s approach, the patient population, the tools and resources developed and/or identified by the Workgroup, actions the Workgroup has taken to date, and the Workgroup’s current and future direction to address the inequities brought to light by COVID-19. Given the multiple events and tasks related to the Workgroup, a timeline of events is shown in Table 1.

**Table 1:** Time of COVID-19 Mitigation Workgroup Events and Activities

Dates	Events/Activities
Early March 2020	Westfield MA COVID-19 outbreak occurs
Late March 2020	Westfield outreach efforts
April 2020	Mitigation team commissioned
April 21, 2020	Mitigation team first meets
April–May 2020	Work on 6 goals
April 2020	Pharmacy, food, travel resources identified
May 1, 2020	First GIS map for high-risk areas completed
Early May 2020	Split to patient-focus and community outreach branches
Early May 2020	Survey of community leaders/influencers
Late May–August 2020	Development and testing of needs assessment tools
June 2020	GIS risk combined with patient level risk
June 25, 2020	Religion and Culture Webinar
June 2020	Receipt of Community Foundation Western MA grant
September 2020	Outreach by community health workers started
November 2020	Revitalize Community Development Corporation joins mitigation outreach for food delivery
August–October 2020	Testing and education events in community
January 2021	Shift to incorporate vaccinations

### The Workgroup

#### Workgroup inception

As the inequities of impact of the COVID-19 pandemic came into clear view, in April 2020 Baystate Medical Center’s leadership convened a Workgroup with the purpose of developing a cohesive and systematic plan to mitigate the impact of COVID-19 on vulnerable patients in our communities, particularly those with significant social needs. We knew that the Workgroup needed to be an interdisciplinary team of leaders across multiple organizations in our system and it was intentionally constructed for these members to bring their expertise, authority, influence, and connections to ensure that we could incisively design and implement a multifaceted approach to a complex problem. Thus, we invited leaders from multiple affiliated organizations in western Massachusetts to join the Workgroup. Baystate Health is a large integrated health system with four community health centers in Springfield. Caring Health Center is a Federally Qualified Health Center (FQHC) that is also located in Springfield. The BeHealthy Partnership is a Medicaid Accountable Care Organization (ACO) comprised of Health New England as the insurer and Baystate Health and Caring Health Center as the care delivery sites. The Public Health Institute of Western Massachusetts is a non-profit organization that facilitates community engagement and collaborative partnerships, conducts community-participatory research and evaluation, and promotes policy advocacy. University of Massachusetts Medical School-Baystate is the western campus of the medical school and is home to the Population-based Urban and Rural Community Health (PURCH) program. Table 2 shows the roles and titles of the Workgroup members we assembled listed in alphabetical order. The Workgroup has met regularly since its inception and has maintained its constituency. Because of the need to distance and time demands of the Workgroup’s membership, the meeting has been on a virtual platform from the start and continues to gather in this way. Of note, incentives for involvement are the criticality of the need to care for our most vulnerable patients, alignment of the objectives to those central to our organizations, and the high level of professional and personal relevance to our positions and roles in our organizations.

**Table 2:** The Roles and Titles of the COVID-19 Mitigation Workgroup

Organizational Role	Workgroup Sub-team
Associate Hospital Epidemiologist, Infectious Disease Division, Baystate Health	Identification
Chair, Department of Medicine, Baystate Health; Chair, Department of Medicine, University of Massachusetts Medical School-Baystate; Co-Chief Medical Officer, BeHealthy Partnership^a^	Identification
Chief Diversity & Inclusion Officer, Baystate Health^a^	Address Needs, Outreach, Needs Assessment
Chief Education Officer, Baystate Health; Senior Associate Dean of Education, University of Massachusetts Medical School -Baystate	Address Needs
Chief, Division of General Medicine and Community Health, Baystate Health^a^	Identification, Needs Assessment, Outreach, Address Needs, Testing
Co-Chief Medical Officer, BeHealthy Partnership	Identification, Needs Assessment, Outreach, Address Needs, Testing
Director, Community Relations, Baystate Health	Outreach
Epidemiologist/GIS Professional^a^	Identification
Executive Director, Public Health Institute of Western MA and Co-Director, BeHealthy Partnership^a^	Needs Assessment, Address Needs, Outreach
Marketing Manager, Baystate Health	Outreach
Population Health Capstone Director, Co-Leader Capstone Scholarship and Discovery Course, University of Massachusetts Medical School – Baystate^a^	Outreach, Testing
Strategic Grant Writer, BeHealthy Partnership and Baystate Health^a^	Outreach
Vice Chair, Clinical Affairs, Department of Medicine and Hospital Epidemiologist, Division of Infectious Diseases, Baystate Health	Identification
Vice President, Diagnostic Services, Baystate Health	Testing
Vice President, Government & Community Relations, Baystate Health	Outreach
Vice President, Marketing & Communications, Baystate Health	Outreach
Vice President, Public Health, Baystate Health	Outreach, Address Needs
Chief Research & Population Health Officer, Caring Health Center (FQHC)^a^	Identification, Needs Assessment, Outreach, Address Needs, Testing

^a^ indicates authors of this report

#### Goal and objectives

In our first meeting on April 21, 2020, we agreed on the goal of developing a cohesive and systematic plan to mitigate the impact of COVID-19 on the most medically vulnerable in our communities, particularly those with significant social needs. Within this goal, we had four categories: identification, outreach, needs assessment, and addressing basic needs. Identification was finding and focusing on the highest need patients. Outreach was engaging patients and communities with the purpose of enhancing our grasp of the broader contexts of patient and community needs, understanding of COVID risks, and beliefs related to COVID, as well as an opportunity for us to deliver education where gaps were identified. Needs assessment was specifically identifying materials and services that would help reduce the risk of contracting or spreading COVID infection. Addressing basic needs was delivering the materials and services based on what we learned through the needs assessments. We then developed a set of objectives and determined the actions required to meet them:

1. Identify vulnerable patients in high-risk geographic areas (identification)
2. Identify material and service needs of patients in high-risk areas through the development and implementation of a needs assessment tool tailored to cultural, linguistic, and religious contexts of the patient population (outreach/needs assessment)
3. Identify pharmacies to provide medication delivery in high-risk areas (address basic needs)
4. Gather information on sources of food delivery, groceries, and/or prepared food in high-risk areas (address basic needs)
5. Gather information on means of travel in high-risk areas (address basic needs)
6. Assess need for COVID-19 testing in high-risk areas (address basic needs)

#### Structure of the Workgroup’s approach

The Workgroup initially was arranged into six subgroups to work on these objectives, making progress between the larger Workgroup meetings. Membership of each subgroup is indicated in Table 2. Within a month, the subgroups completed their individual initial tasks, and the focus of the Workgroup then combined the six elements into two branches. The first branch is patient-oriented, and the other branch focuses on community outreach and engagement. Specifically, the patient-oriented actions are now applying the processes we have created for identification, outreach and assessment of needs, information on available resources, and procurement of needed resources. The community outreach and engagement efforts have involved inquiry among community leaders, engaging religious leaders, and mobile education and testing programs.

### Patient population

We agreed to focus on patients in the BeHealthy Partnership Accountable Care Organization (BHPACO). BHPACO serves approximately 44,000 individuals with MassHealth/Medicaid in Western Massachusetts, with the majority of its members residing in Springfield, MA. Health New England manages the insurance aspect of the partnership. To qualify for Medicaid, one must be under 65 years of age and have a low income or be disabled. We selected the BHPACO population because we know the membership fits the definition of vulnerable in multiple ways (e.g. race/ethnicity, language, poverty), we have a large population in the city of Springfield who are BHPACO members, and data for this group is accessible.

Clinical care for BHPACO members is provided by five community health centers in Springfield, four of which are Baystate Health practices and one which is a Federally Qualified Community Health Center. Baystate Brightwood Health Center has had a presence in the North End of Springfield since 1972, and in their present location since 1996. Brightwood has 16 primary care providers (physicians, nurse practitioners, and physician assistants), 9 resident physicians, and 48 support staff comprised of registered nurses, medical assistants, patient service representatives, community health workers, pharmacists, medical interpreters, and others. Brightwood is known for community engagement and activism in serving patients most in need. Baystate’s High Street location is home to two Health Centers since 1990: Adult and General Pediatrics. In the Adult practice, there are 14 primary care providers (physicians, nurse practitioners, and physician assistants), 45 resident physicians, and 54 support staff. General Pediatrics at High Street consists of 13 primary care providers (physicians, nurse practitioners, and physician assistants), 29 pediatric resident physicians, 32 Medicine-Pediatrics residents, and 44 support staff. Both High Street Community Health Centers are highly focused on medical education, excelling in teaching medical residents, nurse practitioner residents, medical students, and learners in a variety of other disciplines. Baystate Mason Square Neighborhood Health Center has served the Mason Square neighborhood from its present location since 1996; there are 19 primary care providers (physicians, nurse practitioners, and physician assistants), 36 Medicine-Pediatrics resident physicians, and 52 support staff. Mason Square is home to a nationally recognized Medicine-Pediatrics residency program.

Caring Health Center (CHC), established in 1995, is a Section 330 federally qualified community health that serves approximately 19,000 patients per year in 39 languages with nearly half (47%) requiring translation services. CHC provides a complete range of primary, dental, behavioral health and substance use disorder, care coordination, reproductive health, pharmacy, WIC and wellness services. As a Refugee Health Assessment provider in Massachusetts, CHC provides care to a global patient population including Puerto Rico, Bhutan, Iraq, Ukraine, Syria, Somalia, and the Democratic Republic of Congo. CHC has approximately 16–20 medical providers in patient care and leadership roles located across three sites within Springfield, MA. The expanded care teams also include nurse care managers, care coordinators, community health workers, medical interpreters, patient navigators (to support enrollment into insurance programming), pharmacists, and nutritionists to address the primary care needs of the patient while mitigating environmental and social barriers to care. CHC’s CHW program was awarded the 2017 Massachusetts Outstanding CHW Program Award of the Year from the MA Community Health Worker Association, and CHC served as a consultant to the BHPACO to hire, train, and integrate CHWs into the care management teams.

### Tools and resources

Please see Table 3 for a concise list of the tools we developed and used. These are described in detail below.

**Table 3:** Tools Developed by the COVID-19 Mitigation Workgroup

Tool	Purpose
GIS Maps for Geographic Identification of Higher-Risk Areas in Springfield	Identification
Risk Registry (Individual Medical Comorbidity + High Geographic Risk)	Identification
Community Leader/Influencer Semi-Structured Survey	Outreach
Patient Needs Assessment Tool	Outreach
No-contact workflow for delivery of needed materials	Address Needs
COVID information scripts for calls and videos	Outreach

#### Identification

We used suitability analysis—a type of spatial decision analysis also known as multi-criteria evaluation or multi-criteria decision analysis--to quantify the decision-making process when geography is in an important factor [14–17]. A suitability model is derived from a set of input criteria and weights subjectively chosen by the decision-makers. The output from the analysis is a map with a color-coded surface from low to high suitability for a defined purpose. In the current application, the aim was to identify locations in Springfield at greatest risk of spread of COVID-19 and bad outcomes from the infection. We continue to focus on these areas and neighborhoods for increased healthcare outreach, with the aim of prevention and care. We used the following criteria to define risk: density of COVID-19 cases tested at Baystate Health facilities; density of the US census population; density of the BHPACO population; and density of low-income housing. Due to a lack of data at the time, criteria were chosen and weighted according to their perceived relative importance in determining risk by a panel of healthcare professionals involved in clinical care and outreach. The weights we chose are shown in Table 4. Based on the impression of the identification team that low-income housing is a strong proxy for vulnerability, we doubled the weight of this criterion relative to the others.

**Table 4:** Criteria and weighting for Suitability Model

Criteria	Weight
Case density	20%
Census population density	20%
BHPACO member density	20%
Low-income housing density	40%

**Fig. 1:**
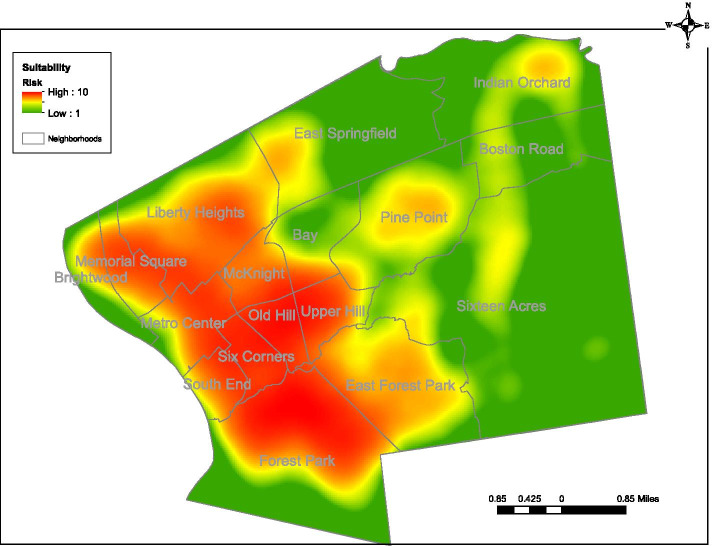
A hypothetical suitability map of Springfield for demonstration purposes

Data for the analysis came from multiple sources. Baystate Health and Pioneer Valley Information Exchange data was used to identify positive COVID −19 tests. We used member files from Health New England to determine the location of BHPACO members. For the location of low-income housing, we used data from the City of Springfield Planning Department. MassGIS (Bureau of Geographic Information) provided the geographic base layers for mapping and the street database for geocoding [18]. The American Community Survey was the source of population estimates using 2018 data, the most recently available [19]. ArcGIS software was used for all geoprocessing and analysis [20]. The earliest map for our suitability model was produced on May 1, 2020, with red representing the highest intensity of likely risk based on the assumptions we loaded into the suitability model and green representing the lowest. Due to data privacy, while our actual maps cannot be included here, Fig. 1 displays a hypothetical suitability map for demonstration purposes.

Many BHPACO patients live in the areas we had identified as high-risk via our GIS maps, so we needed to focus further to be able to ensure we were reaching those who were medically at higher risk in these areas. To account for individual risk based on medical comorbidity, we used claims-based data for patient-level risk factors related to poorer COVID-19 illness outcomes based on information provided on the Centers for Disease Control (CDC) website [21]. These conditions were poorly controlled asthma, severe chronic obstructive pulmonary disease, congestive heart failure with New York Heart Association class III or IV, AIDS, transplant recipient, any cancer with code for chemo or radiation in past month, poorly controlled diabetes, chronic kidney disease on dialysis, and cirrhosis with any code for decompensation. We used these criteria to compile a list of “high-risk” patients with any of these conditions. Due to the lack of severity information from the claims-based data we did not weight any condition or combination of conditions as higher priority with respect to medical comorbidity risk. We then coupled this data with the location risk data from the suitability analysis. All locations in Springfield were scored for risk in the suitability analysis ranging from 10 for highest risk to 1 for lowest risk. These scores were then applied to the street addresses for the high-risk patients. This provided a registry that Community Health Workers (CHWs) from each of our BHPACO community health centers are continuing to use for outreach, as described below.

#### Outreach and needs assessment

Our interdisciplinary outreach and needs assessment team included a social epidemiologist, diversity & inclusion officer, public health advocacy leader, Medicaid ACO co-chief medical officer, a bilingual/bicultural team of four CHWs (one per partner health center), and a consultant partner who supported building the needs assessment into Qualtrics. This team collaboratively developed a culturally and linguistically appropriate needs assessment tool aimed at understanding the COVID-related beliefs, needs, barriers, and risk factors faced by BHPACO patients at high risk (both medically and geographically). The team drew upon CHC experience as a leader in delivery of culturally and linguistically appropriate services and in development and response to emerging needs through public health programming with over two decades of experience collaborating in community responsive research [22]. The goals of the needs assessment were to be dignity-based in terms of the questions asked and the approach for data collection, with the goal of creating a resource delivery program to mitigate COVID transmission and/or exposure, particularly among those with limited capacity to self-isolate (front-line essential workers, multigenerational households, etc.). To protect our CHWs and our patients, CHC’s no-contact delivery workflow was adopted for delivery of needed food and other resources. The needs assessment tool built on the findings from the community leaders/stakeholder surveys including key cultural and faith-based stakeholders as described in the next section.

An initial version of the needs assessment was piloted by trained CHWs with a small group of English and Spanish-speaking patients from two community health centers. Patient responses to and comprehension of the questions combined with feedback from the CHWs about time to administer the tool, ease and flow of the questions, and specific framing of questions informed iterative revisions. After incorporating feedback, the tool was finalized and translated into Spanish. The CHWs administered the needs assessment in their outreach with at-risk patients from the registry to identify immediate clinical and/or service needs as well as specific COVID mitigation material resource needs. The needs assessment was administered with 104 patients (90 English-speakers and 14 Spanish-speakers). Once patterns of need emerged, the needs assessment was no longer administered and CHWs focused on completing no-contact resource delivery of COVID material resources and food to patients isolating with confirmed COVID or awaiting COVID test results. We decided to curtail administering the needs assessment so as not to overburden patients with lengthy phone calls during a time of substantial digital divide.

#### Available resources

Our teams found that commercial pharmacies in these neighborhoods were all willing to deliver to patients and worked with us to implement a delivery plan. Our teams also met with Baystate pharmacy leadership to assure capacity to deliver medications to at-risk ACO members who use Baystate pharmacies. Our team found numerous existing options for obtaining food in/near the target neighborhoods and compiled a resource list for food delivery. We learned that many resources are extant and therefore only require knowledge of their location and coordination to make them available.

#### Procured resources

We received a generous state-funded COVID-19 mitigation grant from the Community Foundation of Western Massachusetts. We used these funds to purchase supplies to respond directly to stated needs in our assessments with COVID-19 care packages: reusable facemasks, personal hand wipes, Environmental Protection Agency approved cleaning supplies for killing the COVID-19 virus, digital thermometers, and digital pulse oximeters. We also purchased room dividers and air mattresses to allow for some level of quarantine in crowded living spaces or multigenerational homes. We were also able to leverage state funding connected to the BHPACO for an intensive 2-month program at the end of 2020 delivering boxes of food to BHPACO members through a partnership with Revitalize Community Development Corporation, a community- based organization. Over 1000 food boxes were delivered to households who were at risk for or recovering from COVID-19. In addition, CHC was able to obtain several COVID funding opportunities to develop expanded testing, a public vaccination site, a mobile health unit and two COVID educational videos series in seven languages by members of health center staff.

#### Delivering based on needs

A designated CHW at each of our BHPACO community health centers (five in total) used the data registry to identify high-risk patients by comorbidity and location risk level and then used the culturally and linguistically appropriate needs assessment tool we created. We developed a no-contact workflow for delivery of needed materials that kept our CHWs and patients safe. Based on the patient’s report, the CHW arranged for no-contact delivery of the needed materials and food. Additionally, we expanded the CHW no-contact delivery workflow to include patients referred by health center providers of patients who tested positive with COVID or awaiting COVID test results. We tracked referrals using our case management documentation system, allowing CHWs to log contacts with patients, patient needs, and delivery of materials, food, and services. CHWs also documented actions in the electronic medical record.

### Community outreach and engagement efforts

#### Outreach

We found that information challenges are particularly acute among certain immigrant populations. In Westfield, MA, a town to the west of Springfield, a COVID-19 outbreak occurred in early March 2020. In response, a team from Baystate Health piloted a program aimed at Westfield’s large Russian-speaking community. To better understand and address the unique needs of this community, a survey was developed and administered to leaders in the Russian community including faith leaders and owners of the local Russian newspaper, grocery store, and deli. Survey respondents were asked questions about themselves and their community. Survey questions included sources of COVID-related information, assessment of the level of understanding of COVID, including common misunderstandings, and whether their community’s culture may affect understanding of and compliance with recommended behaviors to mitigate disease transmission. One strong recommendation was that information be translated into Russian and be culturally adapted based on communication and cultural and religious patterns and beliefs. As a result, a Russian language flyer was developed and made available to all survey participants in both hardcopy (1200 flyers) and electronically. Members of this team joined our Workgroup and their work provided a model for how to reach linguistically and culturally diverse groups in Springfield.

A second interview survey protocol was developed with key learnings from our Westfield experience and Caring Health Center’s extensive prior outreach expertise. The Workgroup sub-team created a culturally responsive outreach survey questionnaire directed to community leaders and key influencers, especially faith-based leaders in the Black and Latinx communities. We tapped into ongoing Baystate community outreach efforts to connect with our identified populations. In early May 2020, 47 community leaders participated in a 45 to 60-min structured interview using the questionnaire to gather information about understanding of disease, sources of knowledge about disease, connection of disease understanding with a cultural/religious lens, medical and behavioral health needs, and social needs. One of the key findings from these interviews was the level of distrust of the healthcare community, from whom the community is most likely to receive information (religious leaders played a huge impact), and the impact of structural racism on trusting healthcare. The findings informed next steps for our continued patient-level and community-level outreach.

A third interview protocol was developed for community health workers to use with at-risk populations in either English or Spanish. This community needs assessment was designed to identify needs for transportation, personal protective devices, cleaning supplies, food and water, medicine, heat/air conditioning, help with utility bills, access to reliable phone and internet services, childcare, and social connections. In addition, respondents were asked about their feelings of stress or anxiety and sadness or loneliness. Where immediate needs were identified, relevant services were offered and provided. Respondents were also asked about their health information sources, living circumstances and ability to effectively isolate at home, safety behavior, how employment status may have affected how they prefer to utilize healthcare, and willingness to take a COVID vaccine. This information proved vital in coordinating and targeting help for those most impacted by the pandemic.

Baystate’s Chief Diversity & Inclusion Officer led a webinar for the community at large called “Religion & Culture and Its Impact on COVID-19.” This event led to initiating two smaller interactive virtual sessions featuring local religious leaders called “Faith Leaders as Public Health Advocates” which is now being explored as an ongoing activity including a speakers’ bureau and connecting religious leaders and congregations with a trusted medical information source. These activities capitalize on our finding that religious leaders are highly influential in their communities and can effectively promote public health and safety messaging in a way that is both authentic and informed. We are also continuing to expand COVID mitigation outreach strategies with the Springfield Housing Authority and other community stakeholder groups. The Public Health Institute of Western Massachusetts, in collaboration with a local cable access TV station, held a virtual Town Hall before Thanksgiving 2020, giving a platform to multi-lingual and culturally diverse physicians and community members to share their prevention strategies for the holidays and beyond. Currently, members of our team are part of the mayor of Springfield’s “Vax Power” group debunking myths and communicating about the importance, risks, and facts for the vaccine roll-out campaign and “Vax Force” group for vaccine delivery.

#### COVID-19 health information

Based on information from the outreach survey to community influencers and leaders, we developed health information materials addressing myths and providing culturally responsive information. In November 2020, our CHWs noted that many patients had questions about how to travel safely for the upcoming holidays. In our context, this referred mostly to travel from Springfield, MA to Puerto Rico to visit extended family. Using CDC guidance along with MA state travel restrictions, we put together a culturally responsive script. The script was a conversation between a community health worker and a physician about ways to stay safe when traveling, along with suggestions about how to stay connected to family without in-person travel. This was also translated into Spanish and video in both English and Spanish. A similar approach has now been applied to COVID vaccination.

#### Mobile education and testing

We have mobilized efforts that were already in play for bringing education and screening to the community. Leaders of the University of Massachusetts Medical School-Baystate Population-based Urban and Rural Community Health (PURCH) program coupled with a mobile care team conducted a number of education and testing events in the community. The locales for these events were linked to areas of risk identified in our suitability analyses. These were cooperative events with community councils, community-based organizations, and the Springfield Housing Authority and resulted in more than 600 individuals being tested for COVID-19. In addition to testing, these events allowed the opportunity for PURCH students and medical staff to provide education about COVID-19. Some of this work has been done as part of MA’s “Stop the Spread” campaign, actively coordinated with the Springfield Department of Health.

## Discussion

We describe a systematic approach applied by our health system for our most vulnerable patients featuring an integrated team focusing on identification of at risk-patients, outreach to both patients and the community, a culturally and linguistically attuned patient needs assessment, and addressing the basic needs of high-risk patients. All of this activity was developed with the purpose of mitigating COVID-19 risk and impact in a population that the literature and our experience told us would be hit hardest because of community demographics, economic status, medical comorbidity, and circumstances. The inequitable impact of COVID-19 on vulnerable populations has been demonstrated by others. Karaye and Horney approached the question of social vulnerability and location; they found that minority status and language conferred greater risk as did social determinants of health such as household composition and transportation [6]. Winskill and colleagues’ work showed greater risk of COVID-19 in vulnerable and disadvantaged populations, in particular because of challenges to social distancing in larger, inter-generational households, and social determinants such as food insecurity [7]. Correa-Agudelo et al examined drivers of COVID-19 mortality in the US and found great risk of COVID-19 related mortality among African American and Latinx populations [8]. Dasgupta and coworkers found that “counties with more social vulnerabilities, particularly those with a higher percentage of racial and ethnic minority residents, high-density housing structures, and crowded housing units, were at higher risk for becoming a COVID-19 hotspot” [9]. A review by Gil et al assessed available information about the impacts of COVID-19 on Hispanic/Latinx communities and found they are disproportionately affected by COVID-19 with respect to cases, hospitalizations, and mortality [10].

Our thinking and action for a COVID-19 mitigation strategy for at-risk communities and community members is not unique. Mesa Viera and colleagues presented a four-quadrant conceptual model specifically with respect to COVID-19 which is similar in many ways to how we had independently conceptualized our efforts [23]. In Mesa Viera’s model, there are four quadrants: vulnerable groups, well-being, preventive measures, and misinformation. Many of these elements were the same concerns driving our focus on our at-risk populations and in turn helping to structure our planning and actions. Others also have engaged in nearly parallel approaches, such as a number of the elements suggested by Kuy and colleagues’ call to action including culturally and linguistically appropriate outreach, food delivery, and use of geospatial mapping [11]. Similarly, Gil et al recommended an approach that also emphasized linguistically and culturally congruent communication and outreach, strong engagement and establishment of trust between community partners and healthcare delivery teams, access to testing, and a focus on data [10]. Karaye and Horney approached the question of social vulnerability and location, reporting that “minority status and language, household composition and transportation, and housing and disability predicted COVID-19 case counts in the U.S” [6]. We considered similar factors in our early models, but they did not substantively change our assessment of risk by location, so we set them aside in favor of the simpler model we developed to augment locational risk factors with individual-level risk factors.

We are limited in this report in that we did not enter this endeavor with a research mindset but rather a need to act quickly yet deliberately to address a crisis. Therefore, we are only now beginning to assess the impact of our efforts. We currently have IRB approval to test the predictive accuracy of our GIS suitability analysis as we now have historical data to examine, and we can assess whether our team’s assumptions for the factors driving geographic risk were the right choices or whether other factors should have been considered. This may be of great value for either the unfortunate circumstance of this pandemic continuing or future public health emergencies. We will be seeking IRB approval to report on our community leader/influence interviews as well as detailed evaluation of our needs assessment tool. We intend to use propensity score matching of individuals with whom we were unable to connect compared with similar patients we did reach in order to determine whether we impacted outcomes such as COVID infection rates. Future activities, in addition to examination of our effectiveness as described, will focus on testing and vaccination including mobile initiatives and readiness to apply our systemic approach as the situation demands. In addition, we acknowledge the major impact of the Digital Divide in the communities we serve and are engaged in a deep, community-wide exploration of how to bridge this divide for our most vulnerable patients.

Now we can look back and consider whether with the benefit of hindsight we may have done things differently. Certainly, the tension about whether to focus on patients within our healthcare system versus engaging in the public health arena was an ongoing concern. Our inclination was to try to address both without going beyond our own resources and without unknowingly duplicating or overlooking efforts by others. While these efforts have been increasingly more collaborative across private and government sectors over time, the lack of specific infrastructure earlier in the pandemic indicates a need for more proactive planning across entities for future public health threats. Our health system was an early adopter of monoclonal antibody treatments, and while members of our Workgroup were also members of the planning team for these treatments, we could have done more to make this treatment more available to our most vulnerable patients. Throughout this process, we were very aware of the Digital Divide in the community we serve. We explored the possibility of providing devices and internet access, but we encountered cost, regulatory, and infrastructure barriers that were beyond our capacity to rapidly or fully address. We are continuing to explore the local drivers of the Digital Divide and how to mitigate them. Lastly, earlier, deeper, and more comprehensive work on vaccine acceptance would have been helpful as now we are laboring to improve the relatively low vaccination rates in our region and particularly among our BHPACO members. Our early emphasis in vaccination delivery was about access as we believed that the biggest challenges our patients would face would be around transportation, language, and ability to schedule online, but it is now clear that with abundant access to vaccinations in place, beliefs and culture are much more at play and harder to change.

Despite not yet being able to report on data and outcomes, what we have learned is broadly applicable and actionable. We found that moving deliberately and engaging community-based leaders and influencers was an important strategy. Building on already-established relationships of Baystate Health was critical. Because of our long-standing work in the city, the mapping validated what we already knew about neighborhoods that would benefit from more attention and resources. Using established relationships helped us respond quickly and more authentically. Combining geographic risk with patient-level comorbidity-driven risk meant that we were able to direct limited resources to those who we are confident needed our support the most. The known areas of geographic risk also direct community outreach efforts. Our work has shifted to using many of the same techniques, such as GIS analyses, applied to vaccination delivery and outreach. We have continued to link our outreach efforts to community-based organizations and groups but have also joined with other local healthcare systems and have shared our lessons learned to strengthen and expand vital collaborations. A comprehensive approach using a multidisciplinary team with substantive influence in and beyond our healthcare system meant we could be effective and that our work in multiple areas was informed by activities in other areas.

## Conclusions

A highly intentional and methodical approach to patient and community outreach with a strong geographic component has led to fruitful efforts in COVID-19 mitigation. Our patient level outreach engages our health center clinical teams, particularly CHWs, and is providing the direct benefit of material and service resources to at-risk patients and their families. Our community efforts leveraged existing relationships and created new partnerships that continue to inform us as a healthcare entity, healthcare employees, and clinical teams so that we can grow and learn in order to authentically build trust and engagement. This important bi-directional learning will help us to become more effective providers and create more opportunities to promote community health. We are now embarking on a systematic approach to evaluating the impacts of our work, supporting vaccine implementation, and supporting our patients and families who have been devastated by COVID-19. We intend to continue disseminating our methods and findings as a template that others can adopt, adapt, and apply in their communities.

## Data Availability

Not applicable.

## Abbreviations

ACO: Accountable Care Organization
BHPACO: BeHealthy Partnership Accountable Care Organization
CDC: Centers for Disease Control
CHC: Caring Health Center
CHW: Community Health Worker
FQHC: Federally Qualified Health Center
MA: Massachusetts
PURCH: Population-based Urban and Rural Community Health

## References

[1] Waisel DB (2013). Vulnerable populations in healthcare. Curr Opin Anaesthesiol.

[2] Stanhope M, Turner LM, Riley P. Vulnerable populations. Preface. Nurs Clin North Am. 2008;43(3):xiii-xvi. doi: 10.1016/j.cnur.2008.05.001. PMID: 18674666. 10.1016/j.cnur.2008.05.001 18674666

[3] Fiscella K, Shin P. The inverse care law: implications for healthcare of vulnerable populations*.* J Ambul Care Manage 2005;28(4):304–12. doi: 10.1097/00004479-200510000-00005. PMID: 16172559. 10.1097/00004479-200510000-00005 16172559

[4] Vulnerable populations: who are they? Am J Manag Care. 2006;12(13 Suppl):S348–52 PMID: 17112321. 17112321

[5] Hurst SA (2008). Vulnerability in research and health care; describing the elephant in the room?. Bioethics..

[6] Karaye IM, Horney JA. The Impact of Social Vulnerability on COVID-19 in the U.S.: An Analysis of Spatially Varying Relationships. Am J Prev Med. 2020;59(3):317–325. doi: 10.1016/j.amepre.2020.06.006. Epub 2020 Jun 26. PMID: 32703701; PMCID: PMC7318979. 10.1016/j.amepre.2020.06.006 PMC7318979 32703701

[7] Winskill P, Whittaker P, Walker P *et al*. Equity in response to the COVID-19 pandemic: an assessment of the direct and indirect impacts on disadvantaged and vulnerable populations in low- and lower middle-income countries. Imperial College London (12-05-2020), doi: 10.25561/78965.

[8] Correa-Agudelo E, Mersha T, Hernandez A, *et al*. Identification of Vulnerable Populations and Areas at Higher Risk of COVID-19 Related Mortality in the U.S. *medRxiv* 2020.07.11.20151563; doi: 10.1101/2020.07.11.20151563. 10.3390/ijerph18084021 PMC8070560 33921217

[9] Dasgupta S, Bowen VB, Leidner A (2020). Association between social vulnerability and a County's risk for becoming a COVID-19 hotspot - United States, June 1-July 25, 2020. MMWR Morb Mortal Wkly Rep.

[10] Gil RM, Marcelin JR, Zuniga-Blanco B (2020). COVID-19 pandemic: disparate health impact on the Hispanic/Latinx population in the United States. J Infect Dis.

[11] Kuy S, Tsai R, Bhatt J (2020). Focusing on vulnerable populations during COVID-19. Acad Med.

[12] Public Health Institute of Western Massachusetts, Springfield Health Equity Report, Looking at Health Through Race and Ethnicity: 2019 Update.

[13] City of Springfield Housing Study, June 2018.

[14] Carver S (1991). Integrating multi-criteria evaluation with geographical information systems. Int'l J of GIS.

[15] Hopkins L (1977). Methods for generating land suitability maps: a comparative evaluation. J of the Am Institute of Planners.

[16] Malczewski J (2006). GIS-based multicriteria decision analysis: a survey of the literature. Int J Geogr Inf Sci.

[17] Pereira J, Duckstein L (1993). A multiple criteria decision-making approach to GE-based land suitability evaluation. Int J Geographical Information Science.

[18] https://www.mass.gov/orgs/massgis-bureau-of-geographic-information. Last accessed 18 August 2021.

[19] https://www.census.gov/programs-surveys/acs. Last Accessed 18 Aug 2021.

[20] ESRI (2011). ArcGIS desktop: release 10.

[21] https://www.cdc.gov/coronavirus/2019-ncov/need-extra-precautions/people-with-medical-conditions.html

[22] Shaw SJ, Korchmaros JD, Huebner Torres C, Totman MS, Lee. (2019). The RxHL study: community responsive research to explore barriers to medication adherence. Health Educ Res.

[23] Mesa Vieira C, Franco OH, Gómez Restrepo C, Abel T (2020). COVID-19: the forgotten priorities of the pandemic. Maturitas..

